# Leisure activity participation and risk of dementia: An 18-year follow-up of the Whitehall II Study

**DOI:** 10.1212/WNL.0000000000010966

**Published:** 2020-11-17

**Authors:** Andrew Sommerlad, Séverine Sabia, Gill Livingston, Mika Kivimäki, Glyn Lewis, Archana Singh-Manoux

**Affiliations:** From the Division of Psychiatry (A.S., G. Livingston, G. Lewis) and Department of Epidemiology and Public Health (S.S., M.K., A.-S.M.), University College London; Camden and Islington NHS Foundation Trust (A.S., G. Livingston, G. Lewis), London, UK; Université de Paris (S.S., A.-S.M.), Inserm U1153, Epidemiology of Ageing and Neurodegenerative Diseases, France; and Clinicum and Helsinki Institute of Life Science (M.K.), University of Helsinki, Finland.

## Abstract

**Objective:**

To test the hypothesis that leisure activity participation is associated with lower dementia risk, we examined the association between participation in leisure activities and incident dementia in a large longitudinal study with average 18-year follow-up.

**Methods:**

We used data from 8,280 participants of the Whitehall II prospective cohort study. A 13-item scale assessed leisure activity participation in 1997–1999, 2002–2004, and 2007–2009, and incidence of dementia (n cases = 360, mean age at diagnosis 76.2 years, incidence rate 2.4 per 1,000 person-years) was ascertained from 3 comprehensive national registers with follow-up until March 2017. Primary analyses were based on complete cases (n = 6,050, n cases = 247) and sensitivity analyses used multiple imputation for missing data.

**Results:**

Participation in leisure activities at mean age 55.8 (1997–1999 assessment), with 18.0-year follow-up, was not associated with dementia (hazard ratio [HR] 0.92 [95% confidence interval 0.79–1.06]), but those with higher participation at mean age 65.7 (2007–2009 assessment) were less likely to develop dementia with 8.3-year follow-up (HR 0.82 [0.69–0.98]). No specific type of leisure activity was consistently associated with dementia risk. Decline in participation between 1997–1999 and 2007–2009 was associated with subsequent dementia risk.

**Conclusion:**

Our findings suggest that participation in leisure activities declines in the preclinical phase of dementia; there was no robust evidence for a protective association between leisure activity participation and dementia. Future research should investigate the sociobehavioral, cognitive, and neurobiological drivers of decline in leisure activity participation to determine potential approaches to improving social participation of those developing dementia.

Participation in leisure activities has benefits for general health and well-being. Given the increasing numbers of people with dementia, there is considerable interest in effective approaches for prevention.^1^ Five of 7 studies in a recent systematic review reported that frequent participation in leisure activities is associated with lower risk of subsequent dementia, suggesting that involvement in such activities may confer cognitive benefit.^2^ Postulated mechanisms are that participation in leisure activities helps build neural pathways and cognitive reserve,^3^ conferring resilience against neuropathologic changes of dementia, reducing harmful stress,^4^ and encouraging a healthier lifestyle.^5^ However, dementia is characterized by a long preclinical phase and most previous positive studies had less than 10 years follow-up, so leisure activities may have been reduced as an early consequence, rather than cause, of subsequent dementia.

Studies with long follow-ups are needed^6^ to address bias due to reverse association, whereby the observed association may be due to the dementia prodrome, which is characterized by reduced leisure activity in the years preceding dementia diagnosis. Furthermore, repeated measures of exposure, in this case leisure activity, allow evaluation of the consistency of associations over time and that of change in exposures in order to provide insight into the direction of associations. In addition, understanding whether particular types of leisure activities have an effect may be informative for guiding specific future prevention approaches.

We therefore aimed to test the hypothesis that leisure activity participation is associated with lower risk of incident dementia in a large longitudinal study over an average 18-year follow-up. Secondary aims were to examine the importance of length of follow-up on the association of activity participation with dementia, associations between specific activities and dementia, and associations between leisure activity change over 10 years and subsequent incident dementia.

## Method

### Study design and participants

The Whitehall II study is an ongoing cohort study, established in 1985 among 10,308 (6,895 men and 3,413 women) London-based civil servants aged between 35 and 55 years who participated in a structured clinical examination and responded to a comprehensive questionnaire at recruitment,^7^ repeated every 5 years. Data on leisure activity participation were first collected during the 1997–1999 study wave, which therefore serves as baseline for the current study, and repeated in the 2002–2004 and 2007–2009 waves; we included all Whitehall II participants who took part in at least one of these waves.

### Measurements

#### Leisure activity participation

Participants reported frequency of participation in 13 leisure activities,^8^ in response to the question “In your spare time, are you involved in any of the following activities? How often have you taken part in these activities in the last 12 months?”

1. Individual occupations (e.g., reading, listening to music)
2. Using a home computer for leisure
3. Courses and education/evening classes
4. Involvement in clubs and organisations, voluntary or official
5. Cultural visits to stately homes, galleries, theatres, cinema, or live music events
6. Positions of office; school governor, councillor, etc.
7. Social indoor games, cards, bingo, chess
8. Gardening
9. Household tasks, e.g., do-it-yourself projects, maintenance, decorating
10. Practical activities, making things with your hands, e.g., pottery, drawing
11. Religious activities/observance
12. Going to pubs and social clubs
13. Visiting friends or relatives

Participants responded to a 4-point Likert scale (never = 0, less often = 1, monthly = 2, weekly = 3), which were summed to yield a total leisure activity scale (scale 0–39). These measures previously showed a positive association with sleep quality^9^ and cross-sectionally with cognitive function.^8^

#### Dementia

Dementia diagnosis was derived from 3 linked electronic health records to March 31, 2017.^10^ National Health Service (NHS) digital hospital episode statistics (HES) and mental health services data (MHSD) include inpatient, emergency department, and outpatient records, including memory clinics, which are the primary UK dementia diagnostic services.^11^ The linked HES/Office of National Statistics mortality data include documented causes of death. Diagnoses are recorded as ICD-10^12^ codes; F00x–F03x, F05.1, and G30x–31.0 indicate any subtype of dementia. These data contain comprehensive records of people with diagnosed dementia in England, where 69% of those estimated to have dementia have a coded diagnosis.^13^ Sensitivity for dementia diagnosis is 78% in HES^14^ and 54% in mortality register^15^ and sensitivity has been increasing since 2006; additional use of MHSD and mortality data is likely to increase sensitivity for dementia diagnosis.

#### Covariates

Sociodemographic and lifestyle factors were measured by self-report; health status was derived from multiple available data (i.e., self-report, structured clinical examination, and health records); and body mass index, blood pressure, fasting glucose, and cognition were assessed in structured clinical examination. Sociodemographic characteristics included sex, ethnicity (White, other ethnicity), and level of education (no formal education, lower secondary, higher secondary, graduate, postgraduate) assessed at study baseline and age, marital status (married, single, divorced, widowed), occupational position based on grade of last employment (professional, managerial, skilled nonmanual, skilled manual, partly skilled, nonskilled), and employment status (employed, retired/unemployed) assessed at all waves.

Health behaviors were derived from questionnaire at all waves: weekly alcohol consumption (0, 1–7, 8–14, >14 units), smoking (never, ex-smoker, current smoker), and hours per week of moderate or vigorous physical activity (log-transformed due to non-normal distribution). Chronic illnesses were derived from a combination of questionnaire, clinical examination, or linked electronic health records at all waves: body mass index (BMI), hypertension (either taking an antihypertensive or having systolic blood pressure ≥141 mm Hg), type 1 or 2 diabetes mellitus (either having previously received diagnosis of diabetes mellitus, taking antidiabetic medication, having fasting plasma glucose ≥7.1 mmol/L, or plasma glucose 2 hours after oral glucose tolerance test ≥11.1 mmol/L), clinically recorded acute stroke (not including transient ischemic attack, as these are often underdiagnosed so may not appear in the electronic health records we used to ascertain these data), and coronary heart disease (from HES). Cognition was assessed by structured clinical examination at the 1997–1999, 2002–2004, and 2007–2009 waves by assessing verbal fluency, short-term verbal memory, and verbal and mathematical reasoning.^16^

### Analytic approach

We first described the characteristics of the whole cohort according to dementia status and baseline leisure activity participation using *t* test and χ^2^ test. We then examined whether key characteristics varied according to nonparticipation in study waves or missing leisure activity data.

#### Association between leisure activity participation and incident dementia

We first calculated dementia incidence rates according to tertiles of leisure activity participation (low leisure activity was 0–13, medium was 14–18, and high was 19–33) and then calculated absolute rate differences between groups at each of the different study waves. Then our primary analysis examined the association between total leisure activity participation at 1997–1999, 2002–2004, and 2007–2009 and subsequent incident dementia using Cox regression, after checking for the proportionality of hazards assumption.^17^ We found no evidence of interaction by sex (*p* = 0.72) so did not stratify our analysis by sex. We censored participants at date of dementia diagnosis, death, or March 31, 2017, whichever came first. We repeated the analysis for each of the 13 activities. Results for the total leisure activity participation score are presented as hazard ratios (HRs) for dementia according to 1 SD higher activity participation. For each activity, they are presented per 1-point increase on the 4-point Likert scale.

We also examined the association between change in leisure activity participation between 1997–1999 and 2007–2009 and risk of incident dementia after 2007–2009. A positive value in the change score indicated decline in activity participation. In this analysis, HRs represent risk ratios of dementia per 1 SD decline in activity participation. In addition, we derived categories of change in leisure activity participation tertiles from 1997–1999 to 2007–2009: remain low (low at 1997–1999 and 2007–2009), remain medium (medium at 1997–1999 and 2007–2009), remain high (high at 1997–1999 and 2007–2009), increasing (low at 1997–1999 and medium or high at 2007–2009, or medium at 1997–1999 and high at 2007–2009), and decreasing (high at 1997–1999 and medium or low at 2007–2009, or medium at 1997–1999 and low at 2007–2009). We repeated our analyses on the association between change in leisure activity participation and dementia using these categorical groups as exposure variable.

Analyses were adjusted for age and sex; then, in addition, for ethnicity, education, occupational position, marital status, and employment status; then for for smoking, alcohol consumption, and physical activity; then also for health conditions (BMI, hypertension, diabetes, coronary heart disease, and stroke). Sex, ethnicity, and education were taken from baseline and other covariates from the time of exposure measurement. For the analysis on change in leisure activity participation between 1997–1999 and 2007–2009 as a continuous variable, models were additionally adjusted for leisure activity participation at 1997–1999 and sequentially for covariates drawn from the 2007–2009 phase.

#### Sensitivity analyses

We conducted several sensitivity analyses to test the robustness of our results. First, to further test the consistency of our findings, we repeated the primary analyses using leisure activity participation data collected at the 2 other study waves (2006 and 2012–2013), using Cox regression adjusted as above. In post hoc analysis, we examined whether the association between leisure activity participation and dementia incidence was similar using repeat assessments of activity participation, corresponding to increasingly shorter follow-up. We used data from the study waves at which we had activity data at 5 yearly intervals (1997–1999, 2002–2004, 2007–2009, 2012–2013) and included interaction terms between activity participation and wave.

To consider the potential for reverse association in greater detail, we then repeated our primary analyses with additional adjustment for cognitive function at exposure measurement, using a global cognitive *z* score generated as described in previous studies.^16^ Then we repeated the analysis using a 5-year washout period whereby we excluded participants who had less than 5 years follow-up due to incident dementia, death, or end of follow-up, adjusted as before. Finally, as missing leisure activity data were associated with older age, female sex, unmarried status, and incident dementia, we repeated the primary analyses using multiple imputation,^18^ using covariates and dementia status, and leisure activity data from all waves, to assess the potential influence of missing data.

All analyses were undertaken using STATA SE version 14; 2-sided *p* < 0.05 was considered statistically significant.

### Standard protocol approvals, registrations, and patient consents

The Whitehall II study was approved most recently by NHS London–Harrow Research Ethics Committee, reference number 85/0938. Written informed consent for participation was obtained at each contact.

### Data availability

Data cannot be made publicly available because of ethics and institutional review board restrictions. Researchers can apply for data access at ucl.ac.uk/whitehallII/data-sharing.

## Results

**Figure 1:**
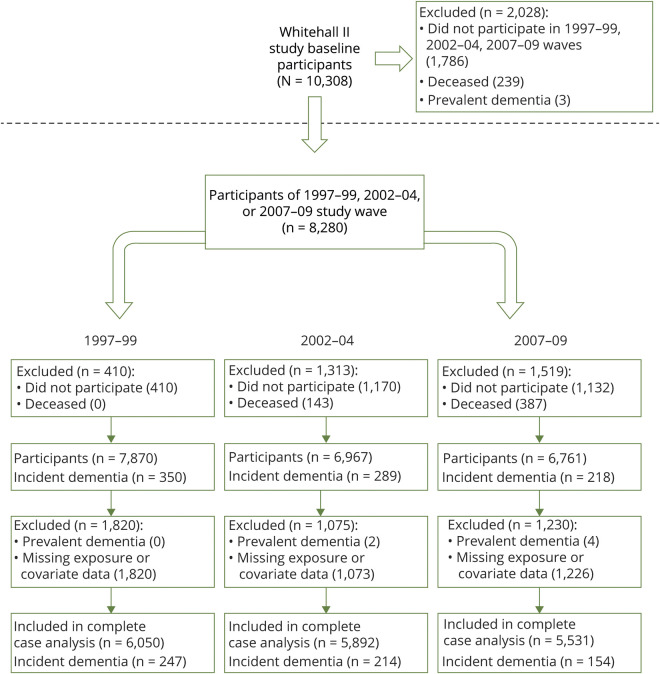
Flow chart of participants in the study (n = 8,280)

Participant flow is summarized in figure 1; 8,280 people participated in 1997–1999, 2002–2004, or 2007–2009, of whom 360 had developed dementia and 1,111 died by March 31, 2017. During 147,774 person-years at risk, 360 incident dementia cases were recorded (incidence 2.4 per 10,000 person-years). The mean age at dementia diagnosis was 76.2 years (SD 5.5, range 58.6–86.0). Full demographic information is available in table 1; 69% of participants were male, 91% were White, and mean age at the start of follow-up (1997–1999) was 55.9 years (SD 6.0, range 44.8–69.2). In univariate analyses, dementia status was associated with baseline sociodemographic factors, alcohol consumption, BMI, chronic illness, and leisure activity participation.

**Table 1:**
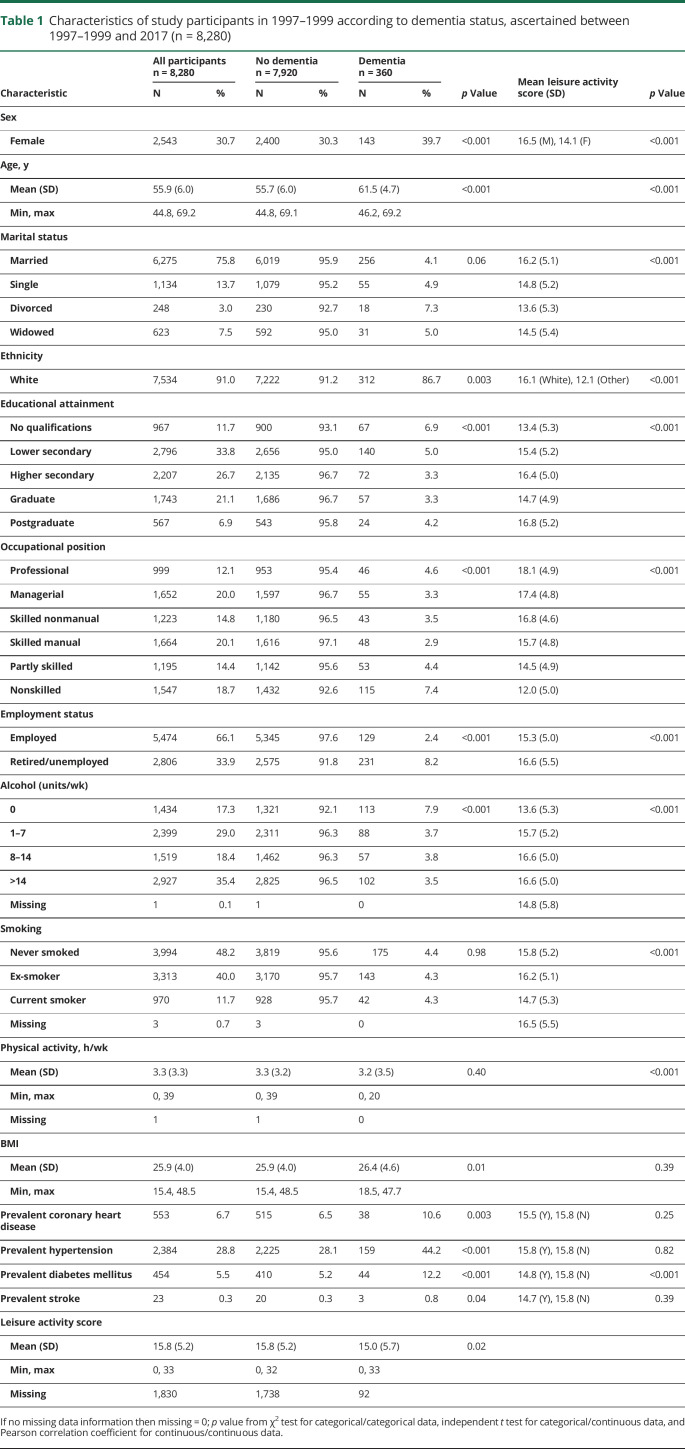

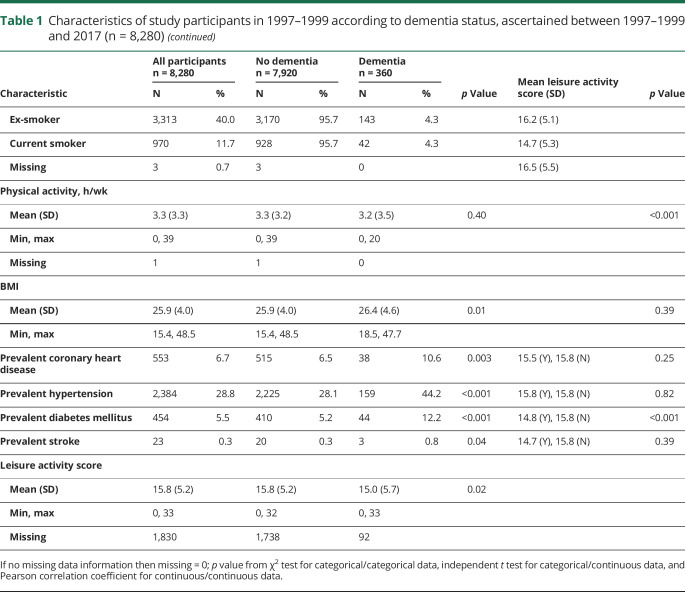
Characteristics of study participants in 1997–1999 according to dementia status, ascertained between 1997–1999 and 2017 (n = 8,280)

### Association between leisure activity participation and incident dementia

Leisure activity participation increased during study follow-up (table 2) from 1997–1999 (mean score 15.8, SD 5.2) to 2007–2009 (18.4, SD 5.3). There was no unadjusted dementia incidence rate difference between tertiles of leisure activity participation in 1997–1999. Compared with an incidence rate of dementia of 2.6 (95% confidence interval [CI] 2.1, 3.2) per 1,000 person-years in those in the lowest tertile of leisure activity participation in 1997–1999, the absolute rate differences per 1,000 person-years were −0.3 (95% CI −1.0 to 0.4) for the intermediate group and −0.6 (−1.3 to 0.1) for the group in the highest tertiles of leisure activity participation. However, dementia incidence rates were lower in the higher tertiles of leisure activity in 2002–2004 and 2007–2009. Compared with the lowest tertiles, absolute rate differences per 1,000 person-years were −1.1 (−2.0, −0.1) in the intermediate and −1.2 (−2.1, −0.3) in the highest tertiles in 2002–2004, and −2.9 (−4.2, −1.5) in the intermediate and −3.8 (−5.1, −2.5) in the highest tertiles in 2007–2009.

**Table 2:**
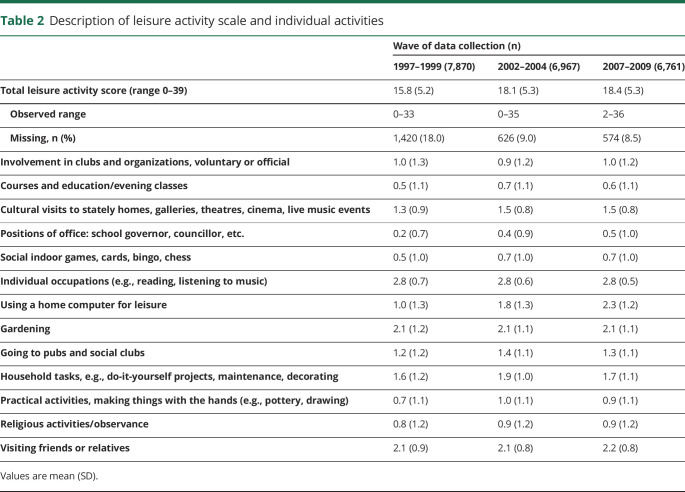
Description of leisure activity scale and individual activities

In fully adjusted Cox regression analyses (table 3), higher leisure activity participation at 1997–1999 or 2002–2004 was not associated with lower risk of dementia (HR per SD higher score 0.92, 95% CI 0.79, 1.06, *p* = 0.24 and 0.88 [0.76, 1.03], *p* = 0.10, respectively) over mean 18.0 and 13.0 years follow-up, respectively. However, there was association between activity participation at 2007–2009 (HR 0.82 [0.69, 0.98], *p* = 0.03) and subsequent dementia with mean 8.3 years follow-up.

**Table 3:**
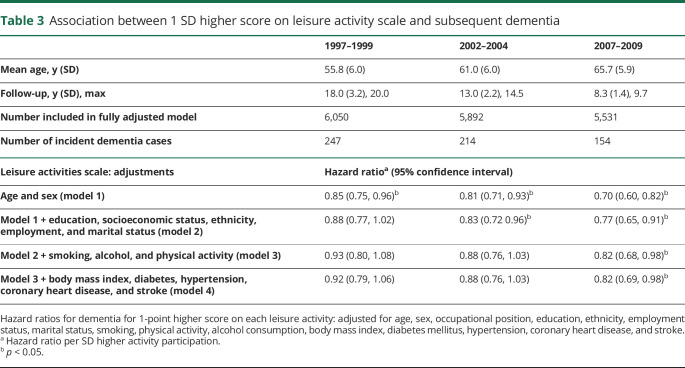
Association between 1 SD higher score on leisure activity scale and subsequent dementia

### Associations between individual activities and subsequent incident dementia

**Figure 2:**
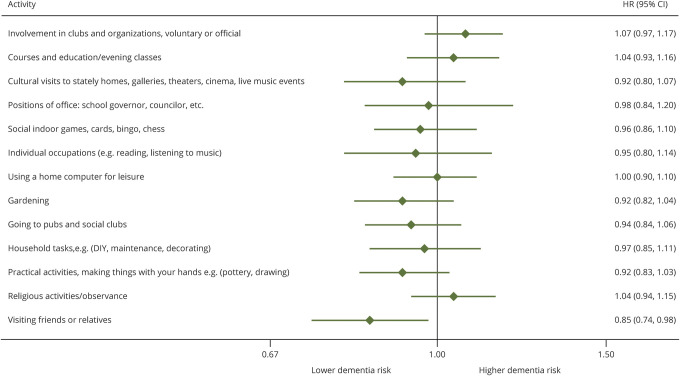
Association of each leisure activity in 1997–1999 with subsequent incident dementia CI = confidence interval; HES = hospital episode statistics; HR = hazard ratio.

Figure 2 shows the association between individual activities at the 1997–1999 study wave and subsequent incident dementia in fully adjusted models; associations at other study phases are in table 4*.* No consistent associations were found as only “visiting friends and relatives” was associated with dementia risk (HR per 1 point increase on activity scale = 0.85 [0.74, 0.98]), but this association was not seen across subsequent study waves. Participation in 4 different activities at the 2007–2009 study wave (positions of office, individual occupations, home computing, and household tasks) was associated with subsequent dementia risk, but these leisure activities were not associated with dementia when drawn from earlier study waves (table 4).

**Table 4:**
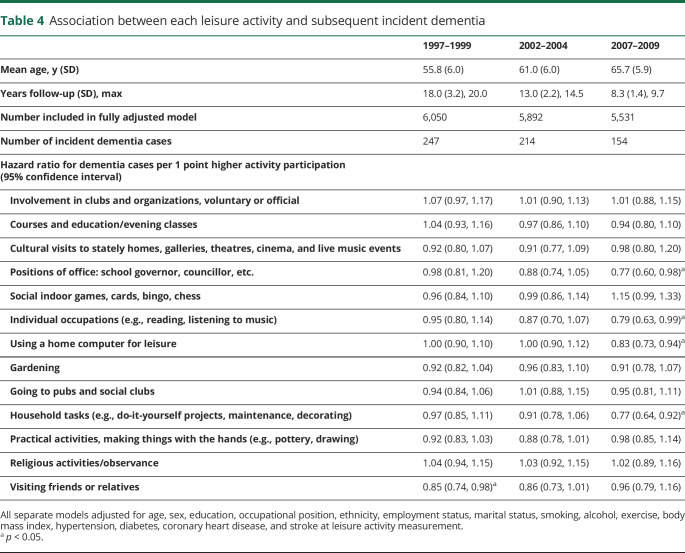
Association between each leisure activity and subsequent incident dementia

### Association between leisure activity change over 10 years and subsequent incident dementia

The mean leisure activity score increased by 2.6 points (SD 4.7, range −15 to +30) from 1997–1999 to 2007–2009. Of the participants who provided data on leisure activity participation at both waves, 820 (17.7%) remained low, 770 (16.6%) remained medium, 892 (19.2%) remained high, 997 (21.5%) increased, and 1,159 (25.0%) decreased participation. For 1 SD decline in leisure activity participation, the HR for incident dementia during the subsequent mean 8.3 years was 1.35 (1.10, 1.66) (table 5). No association was found between categories of change in leisure activity participation and subsequent dementia.

**Table 5:**
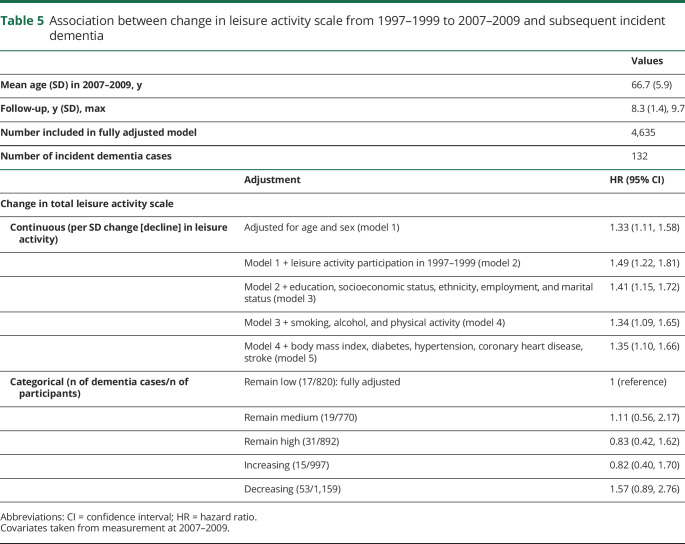
Association between change in leisure activity scale from 1997–1999 to 2007–2009 and subsequent incident dementia

### Sensitivity analyses

Additional analyses of the associations between activity participation at 2006 and 2012–2013 and incident dementia over 10.2 and 4.4 years mean follow-up, respectively, were consistent with the pattern of stronger association when follow-up was shorter. Fully adjusted HR was 0.76 (0.66, 0.89) for the 2006 study wave with mean 10.2 years follow-up, and 0.68 (0.53, 0.86) for the 2012–2013 wave with 4.4 years follow-up. There was evidence for a trend toward lowering of HR in analyses using activity data from later study waves where the assessment of leisure activity was closer to dementia diagnosis (*p* = 0.03).

When we in addition adjusted for baseline cognitive ability, we found no association of leisure activities with incident dementia at any study wave (fully adjusted HR for incident dementia per SD higher activity participation 0.99 [0.84, 1.18] for the 1997–1999 study wave [n dementia cases/n participants = 187/4,984]; HR 0.97 [0.83, 1.13] for 2002–2004 [205/5,747]; and HR 0.92 [0.76, 1.12] for 2007–2009 [133/5,379]). When we applied a 5-year washout period to analyses, we also found no association between leisure activities at any study wave and incident dementia (HR 0.93 [0.80, 1.08] for 1997–1999 [241/5,942]; HR 0.92 [0.78, 1.08] for 2002–2004 [196/5,759]; and HR 0.89 [0.71, 1.12] for 2007–2009 [92/5,292]).

Use of multiple imputation to account for missing data on leisure activity and covariates found results consistent with our primary analyses. For the association between total leisure activities and subsequent incident dementia, the fully adjusted HR at 1997–1999 was 0.90 (0.78, 1.02) (360 dementia cases/8,280 participants); for 2002–2004 HR 0.87 (0.77, 0.99) (349/8,081); for 2007–09 HR 0.78 (0.68, 0.90) (299/7,772). In models using multiple imputation, no specific leisure activities were consistently associated with incident dementia. For change in leisure activities from 1997–1999, HR for incident dementia per SD decline in activity participation was 1.38 (1.20, 1.59) in 7,772 participants with 299 dementia cases. Participants in the “decreasing” category had elevated dementia risk compared to those who remained low (HR 1.71 [1.10, 2.67]).

## Discussion

In this large longitudinal study, participation in leisure activities at mean age 56 years was not associated with incidence of dementia over the subsequent 18 years. Associations were only evident when leisure activity was assessed at older ages, with less than 10 years between assessment of leisure activities and diagnosis of dementia. Decline in leisure activity participation over 10 years was associated with subsequent elevated risk of dementia. No consistent associations were found for participation in specific types of leisure activities. Taken together, these results do not support the hypothesis that leisure activity participation can lower dementia risk, but suggest instead that reduction in activity participation is an indication of possible prodromal dementia.

Our findings contradict the conclusions of previous studies,^19–23^ which reported associations between either a composite measure of leisure activities or specific activities and dementia risk and therefore suggested that activity participation may protect from dementia risk. Apart from one exception,^24^ these studies have had shorter follow-up than our study. A 2016 systematic review included 7 studies in 3 separate meta-analyses according to the analytic methodology of the studies, and each of these meta-analyses found significant associations of higher activity participation with lower dementia risk.^2^ Five of 7 studies reported significant associations but they had less than 6 years between measurement of leisure activities and dementia ascertainment. Two remaining studies had 9^25^ and 12^26^ years of follow-up and they reported null findings in regard to association with dementia. Another study reported association between leisure activities and incident dementia with less than 5 years, but no association with a follow-up greater than 5 years.^27^ Subsequent studies of social engagement with 3 years^28^ and cultural engagement with 10 years^29^ interval between activity measurement and dementia ascertainment also found positive associations between more frequent participation in activities and lower dementia risk. It therefore seems likely that in studies with less than 9 years follow-up or without sufficient washout period, associations found between leisure activity participation and dementia risk are likely due to reverse association. A notable study based on a long follow-up (44 years) of 800 Swedish women reported a binary variable generated from 5 cognitive activity domains to be associated with incident dementia in analyses adjusted for age, physical activity, smoking status, and socioeconomic status but not for education.^24^ The different populations studied, smaller range of activities assessed, and heterogeneity in confounder adjustment may partly explain the differences in findings.

Given the inconsistency in findings as a function of the period of follow-up, we used repeat measures of participation in leisure activities to examine how the length of follow-up affected findings. The underlying assumption is that leisure activities assessed sufficiently long before dementia onset is unlikely to be biased by reverse association. Our results show associations to emerge using the 2002–2004 measure of leisure activities when mean follow-up was 8.3 years and mean age at leisure activity assessment was 65.7 years, and associations strengthened at successive study waves when follow-up was even shorter. Our results cannot rule out the possibility that leisure activity participation after 65 years confers protection against dementia, or that lack of leisure activity or reduction in such activity at a vulnerable time leads to people being more likely to develop dementia. However, there is no compelling mechanism to explain this interpretation of the results given the known long preclinical period of dementia.

Furthermore, the associations between leisure activities at older ages and dementia in our study were attenuated in 2 sensitivity analyses aiming to consider the robustness of findings against bias due to reverse association: (1) when we in addition imposed a washout period of 5 years, thereby removing the potentially biasing effect of a small number of study participants developing dementia in the first 5 years after activity assessment; and (2) when we adjusted for cognitive function at the time of exposure measurement, thereby taking into account cognitive decline in the early dementia prodrome.^30^ These findings, together with our sensitivity analysis indicating an effect of study wave on the association between activity participation and dementia risk, suggest that the protective associations found at later study waves were likely to be due to reverse association. This interpretation is strengthened by our finding that decline in leisure activity participation from age 56–66 years is associated with elevated dementia risk.

Diagnostic criteria specify that dementia be diagnosed when cognitive decline is sufficient to “impair activities of daily living.”^12^ However, early neuropathologic changes of neurodegenerative dementias occur up to 25 years before symptoms are detected^31^ and cognitive symptoms precede dementia diagnostic threshold by approximately 12 years.^32^ It is plausible that decline in leisure activities precedes clinically diagnosed dementia by around 10 years due to the prodromal emergence of dementia symptoms and is in accordance with our findings that 10-year decline in leisure activity participation predicts incident dementia. Neuropsychiatric symptoms including apathy are common in mild dementia and frequently precede dementia onset^33–36^ and may inhibit activity participation. Social cognitive impairments such as stubbornness, lack of concern for others, or emotional control difficulties are common features of mild dementia, possibly due to disruption of amygdala and frontal cortex networks.^37^ These emerging social cognitive changes are related to level of dependence^38^ and may disrupt the social relationships required to participate in activities with others.

Early symptoms of dementia related to social function are frequently misattributed by people with dementia and their family as about choice or personality,^39^ meaning that they may not be supported to maintain activity participation. In addition, cognitive decline is frequently accompanied by physical illness due to shared etiologic pathways,^40^ cognitive difficulties leading to neglect of physical health care,^41^ or physical illness causing neuropathologic damage.^42^ Our finding of a potential prodromal decline in leisure activity participation adjusted for chronic illness and health behaviors supports the notion that multiple mechanisms may underlie effects of preclinical dementia on social participation.

We found no specific activities to be consistently associated, throughout successive study waves, with elevated dementia risk. The only activity that at baseline was associated with subsequent dementia risk was “visiting friends and relatives,” which is consistent with our previous findings in the Whitehall study that more frequent contact with friends and relatives was associated with dementia risk with a 15-year follow-up.^43^ In the present study, the association was no longer statistically significant at subsequent study waves, possibly because a single item was used to assess social contact frequency compared to the more detailed questions in our previous study.^43^

This study's longer follow-up than any previous study examining the association between leisure activity participation and incident dementia and our repeated measurements of activity participation allowed us to examine the potential of reverse association more thoroughly than previously possible. However, our study has limitations. Ascertainment of dementia from electronic health records, rather than through standardized assessment of all study participants, misses undiagnosed cases (22% for HES, 1 of 3 databases used in this study), which are more likely to be mild cases, and could result in bias if missed or delayed diagnostic recording were associated with leisure activity participation. This is plausible as those with lower leisure activity participation may have fewer contacts to encourage health-seeking behavior or lack an informant to give accurate information in clinical settings, although, to our knowledge, no studies have reported association of leisure activity participation with diagnostic sensitivity and results from studies examining other aspects of social participation have been variable.^14,44,45^ The electronic health records also do not accurately tell us the time of symptom onset, which is typically around 3 years earlier than diagnosis,^46,47^ meaning that the prodromal leisure activity decline would be of shorter duration than the 8 years we have identified. Furthermore, health records do not contain comprehensive information about dementia subtype, meaning that we were unable to consider associations between activity participation and particular forms of dementia. However, the databases used for dementia ascertainment cover the predominant UK diagnostic settings and using electronic health records ensures analysis on all participants rather than only those who agree to an in-person assessment, thereby reducing risk of attrition bias.

The range of leisure activities was comprehensive but not exhaustive; some participants may have taken part in other beneficial activities such as watching football or visiting libraries, which were not covered in the questionnaire. Furthermore, data were available only on frequency of participation and other aspects such as duration, intensity, and quality of involvement in these activities were not available, although they would be expected to be linked to frequency. In addition, we were unable to distinguish whether people engaged in specific activities once or many times over a week. The use of self-report allowed repeated assessment of frequency of activity participation and, while self-report is susceptible to measurement error, the fact that our assessments took place so long before dementia onset means that systematic bias in reporting is unlikely.

The study population of predominantly White, male, London-based civil servants may limit generalizability but our sample did include people from a wide range of socioeconomic backgrounds. Loss to follow-up was more likely to occur in older, female, unmarried people and those who went on to develop dementia, but results using multiple imputation to account for missing data due to attrition or nonresponse were consistent with our primary analyses, suggesting that attrition is not an important source of bias for our findings. We combined type 1 and type 2 diabetes mellitus as a single covariate, which have potentially different outcomes, but the majority of the diabetes cases (85%) in our study were diagnosed after study baseline when participants were in midlife, implying that these are type 2 diabetes. Finally, unmeasured confounders may have affected our results as is always the case in observational studies; we have performed several sensitivity analyses and their results are in line with the main analyses.

Whereas leisure activity may benefit mental and physical health, we failed to find evidence that activity participation in midlife would protect against the development of dementia. These findings do not question the importance of leisure activities for general health and well-being; the conclusions drawn in this study are specifically for prevention of dementia. Considering the challenges of conducting randomized controlled trials of midlife lifestyle modifications to reduce dementia risk, the examination of potential risk factors using cohort studies with sufficiently long follow-up to reduce risk of reverse association bias is essential to guide future trials with greater chance of success. There is currently no clear evidence suggesting that modification of leisure activity participation is a priority target for dementia prevention trials.

Our novel finding of association of dementia with activity decline and the timing of this decline suggests that changes in leisure activity participation may be a prodromal feature of dementia, which is consistent with retrospective accounts of decline in participation in activities preceding dementia onset. There should therefore be awareness among clinicians that those who decrease leisure activities in the absence of other causes might be developing dementia.

Future research should aim to characterize the timing of activity decline in relation to other symptoms in greater detail. This may require more accurate methods for measuring activity participation than self-report, which may be inconsistent in people with memory problems, so technological approaches to in vivo measurement^48^ should be evaluated, and dementia status ascertainment should aim to accurately clarify time of dementia onset. Furthermore, understanding of the reasons for social decline is limited, so more detailed assessment of sociobehavioral, cognitive, and neurobiological correlates of social decline in cognitive disorders may elucidate disease processes and identify modifiable risk factors for social decline. These could be targeted in future research aiming to improve social engagement and maximize quality of life for people with dementia and their families.

## Glossary

BMI: body mass index
CI: confidence interval
HES: hospital episode statistics
HR: hazard ratio
ICD-10: International Classification of Diseases–10
MHSD: mental health services data
NHS: National Health Service
